# Personalized prediction of overall survival in patients with AML in non‐complete remission undergoing allo‐HCT

**DOI:** 10.1002/cam4.3920

**Published:** 2021-06-16

**Authors:** Shigeki Hirabayashi, Ryuji Uozumi, Tadakazu Kondo, Yasuyuki Arai, Takahito Kawata, Naoyuki Uchida, Atsushi Marumo, Kazuhiro Ikegame, Takahiro Fukuda, Tetsuya Eto, Masatsugu Tanaka, Atsushi Wake, Junya Kanda, Takafumi Kimura, Ken Tabuchi, Tatsuo Ichinohe, Yoshiko Atsuta, Masamitsu Yanada, Shingo Yano

**Affiliations:** ^1^ Department of Hematology and Oncology Kyoto University Graduate School of Medicine Kyoto Japan; ^2^ Department of Biomedical Statistics and Bioinformatics Kyoto University Graduate School of Medicine Kyoto Japan; ^3^ Department of Transfusion Medicine and Cell Therapy Kyoto University Hospital Kyoto Japan; ^4^ Department of Hematology Hyogo Prefectural Amagasaki General Medical Center Amagasaki Japan; ^5^ Department of Hematology Federation of National Public Service Personnel Mutual Aid Associations Toranomon Hospital Tokyo Japan; ^6^ Hematology Division Tokyo Metropolitan Cancer and Infectious Diseases Center, Komagome Hospital Tokyo Japan; ^7^ Division of Hematology, Department of Internal Medicine Hyogo College of Medicine Nishinomiya Japan; ^8^ Department of Hematopoietic Stem Cell Transplantation National Cancer Center Hospital Tokyo Japan; ^9^ Department of Hematology Hamanomachi Hospital Fukuoka Japan; ^10^ Department of Hematology Kanagawa Cancer Center Yokohama Japan; ^11^ Department of Hematology, Federation of National Public Service Personnel Mutual Aid Associations Toranomon Hospital Kajigaya Kawasaki Japan; ^12^ Preparation Department Japanese Red Cross Kinki Block Blood Center Osaka Japan; ^13^ Department of Pediatrics Tokyo Metropolitan Cancer and Infectious Disease Center, Komagome Hospital Tokyo Japan; ^14^ Tokyo Cancer Registry, Bureau of Social Welfare and Public Health Tokyo Metropolitan Government Tokyo Japan; ^15^ Department of Hematology and Oncology Research Institute for Radiation Biology and Medicine, Hiroshima University Hiroshima Japan; ^16^ Japanese Data Center for Hematopoietic Cell Transplantation Nagoya Japan; ^17^ Department of Healthcare Administration Nagoya University Graduate School of Medicine Nagoya Japan; ^18^ Department of Hematology and Cell Therapy Aichi Cancer Center Nagoya Japan; ^19^ Division of Clinical Oncology and Hematology, Department of Internal Medicine The Jikei University School of Medicine Tokyo Japan

**Keywords:** acute myeloid leukemia, hematopoietic stem cell transplantation, nomogram, non‐complete remission, web application

## Abstract

Allogenic hematopoietic stem cell transplantation (allo‐HCT) is the standard treatment for acute myeloid leukemia (AML) in non‐complete remission (non‐CR); however, the prognosis is inconsistent. This study aimed to develop and validate nomograms and a web application to predict the overall survival (OS) of patients with non‐CR AML undergoing allo‐HCT (cord blood transplantation [CBT], bone marrow transplantation [BMT], and peripheral blood stem cell transplantation [PBSCT]). Data from 3052 patients were analyzed to construct and validate the prognostic models. The common significant prognostic factors among patients undergoing allo‐HCT were age, performance status, percentage of peripheral blasts, cytogenetic risk, chemotherapy response, and number of transplantations. The conditioning regimen was a significant prognostic factor only in patients undergoing CBT. Compared with cyclophosphamide/total body irradiation, a conditioning regimen of ≥3 drugs, including fludarabine, with CBT exhibited the lowest hazard ratio for mortality (0.384; 95% CI, 0.266–0.554; *p *< 0.0001). A conditioning regimen of ≥3 drugs with CBT also showed the best leukemia‐free survival among all conditioning regimens. Based on the results of the multivariable analysis, we developed prognostic models showing adequate calibration and discrimination (the c‐indices for CBT, BMT, and PBSCT were 0.648, 0.600, and 0.658, respectively). Our prognostic models can help in assessing individual risks and designing future clinical studies. Furthermore, our study indicates the effectiveness of multi‐drug conditioning regimens in patients undergoing CBT.

## Graphical abstract

Allogenic hematopoietic stem cell transplantation (allo‐HCT) is the standard treatment for acute myeloid leukemia (AML) in non‐complete remission (non‐CR); however, the prognosis is inconsistent. We developed and validated prognostic models (nomograms and web application) to predict the overall survival of patients with non‐CR AML undergoing allo‐HCT (cord blood transplantation, bone marrow transplantation and peripheral blood stem cell transplantation) using a large cohort. The web application is available at https://JSHCT‐AMLWG.shinyapps.io/Predict‐OS‐nonCR‐AML‐post‐HCT/.

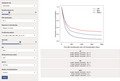

## 1 INTRODUCTION

The prognosis of acute myeloid leukemia (AML) in non‐complete remission (non‐CR) is poor and poses a challenge with respect to the selection of the optimal treatment for patients. Approximately 10%–20% of patients with refractory or relapsed AML exhibit long‐term survival.^1^, ^2^, ^3^, ^4^ Although chimeric antigen receptor T‐cell therapy^5^ and several targeted therapies using FLT3 inhibitors,^6^ IDH1/IDH2 inhibitors,^7^, ^8^ and CD33 antibodies^9^ have been developed, survival outcomes have not been sufficiently improved. Consequently, allogeneic hematopoietic stem cell transplantation (allo‐HCT) remains the most effective treatment to cure refractory or relapsed AML. Recently, it was reported that for acute leukemia or myelodysplastic syndrome, patients with minimal residual disease (MRD) who underwent cord blood transplantation (CBT) showed a more favorable prognosis than those who underwent bone marrow transplantation (BMT) or peripheral blood stem cell transplantation (PBSCT).^10^ Despite the emerging importance of CBT in hematological malignancies with MRD, no large scale studies have been conducted on CBT in patients with AML in non‐CR.

Here, we aimed to identify the prognostic factors and to develop and validate nomograms^11^, ^12^ and a web application for predicting the overall survival (OS) of patients with AML in non‐CR undergoing allo‐HCT, including CBT. Furthermore, we constructed and evaluated prognostic models for BMT and PBSCT. Therefore, our models can simultaneously simulate the prognosis of CBT, BMT, and PBSCT as per the clinicopathological characteristics of each patient and can be helpful in selecting an optimal treatment.

## 2 MATERIAL AND METHODS

### 2.1 Study design and population

**FIGURE 1:**
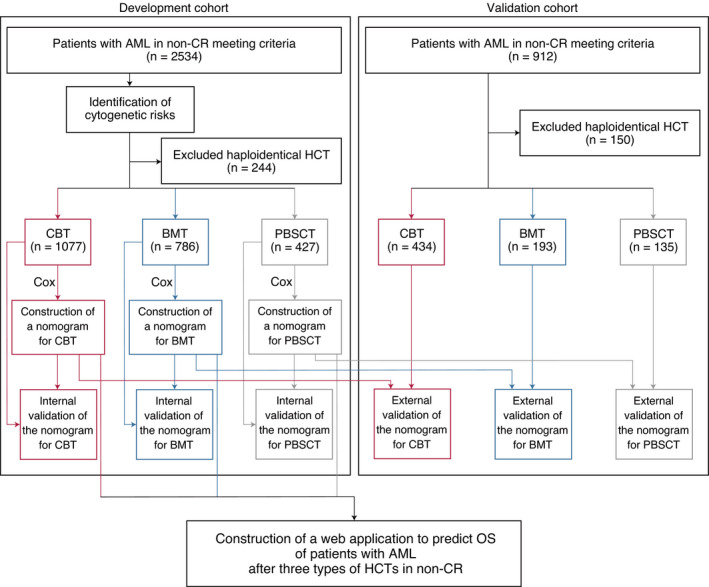
Study design. AML, acute myeloid leukemia; BMT, bone marrow transplantation; CBT, cord blood transplantation; Cox, Cox regression analysis; HCT, hematopoietic cell transplantation; Non‐CR, non‐complete remission; OS, overall survival; PBSCT, peripheral blood stem cell transplantation

In this multicenter, retrospective cohort study, three nomograms and a web application were developed to predict the OS of patients with AML in non‐CR undergoing single‐unit CBT, BMT, and PBSCT. We included consecutive patients undergoing allo‐HCT with AML aged ≥16 years who had ≥5% blasts in the bone marrow or who had ≥20% blasts in the peripheral blood at transplantation. We excluded patients who underwent HCT within 90 days of the last HCT and those who had missing data for potential predictors. We retrieved the data for HCT outcomes from patients at the Transplant Registry Unified Management Program (TRUMP)^13^, ^14^, ^15^ across >300 transplant centers in Japan. The data of patients who underwent allo‐HCT between 2000 and 2014 were used to develop the prognostic models; the data of patients who underwent haploidentical transplantation were excluded. To validate the constructed models, we analyzed the data of patients who underwent allo‐HCT between 2015 and 2016. Figure 1 shows the design of our study.

### 2.2 Variables of interest

Data on clinical outcomes and patient characteristics were retrieved from the registry database. Based on previous reports, we selected the following potential predictors (Tables 1, 2, 3): age of the recipient at transplantation,^1^, ^10^, ^16^, ^17^, ^18^, ^19^, ^20^ sex,^10^, ^16^, ^17^, ^18^, ^19^, ^20^ Eastern Cooperative Oncology Group (ECOG) performance status (PS) at transplantation,^16^, ^17^, ^18^, ^19^ hematopoietic cell transplantation‐comorbidity index (HCT‐CI),^16^, ^17^, ^19^, ^21^ percentage of blasts in the peripheral blood,^18^ French–American–British (FAB) classification,^1^, ^22^ cytogenetics,^1^, ^10^, ^17^, ^18^, ^20^, ^23^ response to chemotherapy,^1^, ^10^, ^18^ disease status,^17^ year of transplantation,^10^, ^17^, ^18^, ^19^ number of transplantations,^1^, ^24^, ^25^ donor type,^17^, ^18^ human leukocyte antigen (HLA) compatibility,^10^, ^16^, ^18^, ^19^ total number of nucleated cells in the cord blood (only for CBT),^19^ conditioning regimen,^16^, ^17^, ^18^, ^19^ prophylaxis for graft versus host disease,^10^, ^18^, ^19^ and use of anti‐thymocyte globulin.^16^

**TABLE 1:** Characteristics of patients who underwent cord blood transplantation

	Development cohort	Validation cohort	
	(*n* = 1077); *n* (%) or median [IQR]	(*n* = 434); *n* (%) or median [IQR]	*p*
Age (years)^a^	56 [44, 64]	57 [45, 65]	0.112
Sex			0.415
Female	427 (39.6)	162 (37.3)	
Male	650 (60.4)	272 (62.7)	
ECOG performance status			<0.001
0	246 (22.8)	116 (26.7)	
1	520 (48.3)	243 (56.0)	
2	205 (19.0)	55 (12.7)	
3	81 (7.5)	17 (3.9)	
4	25 (2.3)	3 (0.7)	
HCT‐CI			0.528
0	461 (42.8)	189 (43.5)	
1–3	470 (43.6)	179 (41.2)	
4–6	129 (12.0)	55 (12.7)	
≥7	17 (1.6)	11 (2.5)	
Peripheral blasts (%)^a^	12.0 [1.40, 46.0]	13.0 [2.00, 50.0]	0.669
FAB classification			0.59
M0	88 (8.2)	27 (6.2)	
M1–2	528 (49.0)	209 (48.2)	
M4–5	174 (16.2)	73 (16.8)	
M6	67 (6.2)	36 (8.3)	
M7	15 (1.4)	5 (1.2)	
Other	205 (19.0)	84 (19.4)	
Cytogenetic risk^b^			0.025
Favorable	618 (57.4)	226 (52.1)	
Intermediate	324 (30.1)	131 (30.2)	
Poor	135 (12.5)	77 (17.7)	
Response to chemotherapy			<0.001
Primary induction failure	438 (40.7)	187 (43.1)	
Duration of first CR, <6 month	205 (19.0)	124 (28.6)	
Duration of first CR, ≥6 month	275 (25.5)	50 (11.5)	
No treatment before transplantation	159 (14.8)	73 (16.8)	
Disease status			0.856
De novo AML	959 (89.0)	385 (88.7)	
Secondary AML	118 (11.0)	49 (11.3)	
Year of transplantation			<0.001
2000–2010	422 (39.2)	0 (0.0)	
2011–2012	313 (29.1)	0 (0.0)	
2013–2014	342 (31.8)	0 (0.0)	
2015–2016	0 (0.0)	434 (100.0)	
Number of transplantations			1
1	828 (76.9)	334 (77.0)	
≥2	249 (23.1)	100 (23.0)	
Donor type			1
Related	0 (0.0)	0 (0.0)	
Unrelated	1077 (100.0)	434 (100.0)	
HLA compatibility			0.089
Match	70 (6.5)	18 (4.1)	
Mismatch	1007 (93.5)	416 (95.9)	
Total number of nucleated cells in the cord blood per body weight – 10^7^/kg^a^	2.65 [2.30, 3.20]	2.66 [2.28, 3.26]	0.947
Conditioning regimen			<0.001
CY+TBI	43 (4.0)	12 (2.8)	
BU+CY	24 (2.2)	7 (1.6)	
CA+CY+TBI	138 (12.8)	47 (10.8)	
FLU+(BU or MEL)	415 (38.5)	106 (24.4)	
FLU+(BU or MEL)+(BU, MEL, CA, or CY)	358 (33.2)	242 (55.8)	
Other regimen	99 (9.2)	20 (4.6)	
GVHD prophylaxis			<0.001
CSA+MTX	168 (15.6)	53 (12.2)	
TAC+MMF	365 (33.9)	201 (46.3)	
TAC+MTX	267 (24.8)	114 (26.3)	
CSA+MMF	34 (3.2)	6 (1.4)	
Other	243 (22.6)	60 (13.8)	
Use of ATG			0.265
No	1048 (97.3)	427 (98.4)	
Yes	29 (2.7)	7 (1.6)	
Treatment for AML after transplantation			<0.001
No	908 (84.3)	341 (78.6)	
Yes	153 (14.2)	93 (21.4)	
Missing	16 (1.5)	0 (0.0)	

Note

*p*‐values were calculated using Fisher's exact test or Wilcoxon Mann–Whitney test based on the categorical or continuous variables.

Abbreviations: AML, acute myeloid leukemia; ATG, anti‐thymocyte globulin; BU, busulfan; CA, cytarabine; CR, complete remission; CSA, cyclosporine; CY, cyclophosphamide; FAB, French‐American‐British; FLU, fludarabine; GVHD, graft versus host disease; HCT‐CI, hematopoietic cell transplantation‐comorbidity index; IQR, interquartile range; MEL, melphalan; MMF, mycophenolate mofetil; MTX, methotrexate; TAC, tacrolimus; TBI, total‐body irradiation.

^a^ Continuous variable.

^b^ Cytogenetic risk determined by this study.

**TABLE 2:** Characteristics of patients who underwent bone marrow transplantation

	Development cohort	Validation cohort	
	(*n* = 786); *n* (%) or median [IQR]	(*n* = 193); *n* (%) or median [IQR]	*p*
Age (years)^a^	53 [42, 60]	56 [45, 63]	0.016
Sex			0.508
Female	292 (37.2)	77 (39.9)	
Male	494 (62.8)	116 (60.1)	
ECOG performance status			0.274
0	278 (35.4)	84 (43.5)	
1	354 (45.0)	79 (40.9)	
2	108 (13.7)	20 (10.4)	
3	36 (4.6)	7 (3.6)	
4	10 (1.3)	3 (1.6)	
HCT‐CI			0.37
0	389 (49.5)	85 (44.0)	
1–3	321 (40.8)	86 (44.6)	
4–6	66 (8.4)	21 (10.9)	
≥7	10 (1.3)	1 (0.5)	
Peripheral blasts (%)^a^	6.05 [0.50, 30.0]	3.00 [0.00, 20.0]	0.019
FAB classification			0.016
M0	74 (9.4)	15 (7.8)	
M1‐2	386 (49.1)	83 (43.0)	
M4‐5	156 (19.8)	29 (15.0)	
M6	63 (8.0)	26 (13.5)	
M7	18 (2.3)	8 (4.1)	
Other	89 (11.3)	32 (16.6)	
Cytogenetic risk^b^			0.142
Favorable	440 (56.0)	100 (51.8)	
Intermediate	247 (31.4)	58 (30.1)	
Poor	99 (12.6)	35 (18.1)	
Response to chemotherapy			< 0.001
Primary induction failure	362 (46.1)	90 (46.6)	
Duration of first CR, <6 month	167 (21.2)	64 (33.2)	
Duration of first CR, ≥6 month	169 (21.5)	19 (9.8)	
No treatment before transplantation	88 (11.2)	20 (10.4)	
Disease status			0.074
De novo AML	691 (87.9)	160 (82.9)	
Secondary AML	95 (12.1)	33 (17.1)	
Year of transplantation			<0.001
2000–2010	352 (44.8)	0 (0.0)	
2011–2012	216 (27.5)	0 (0.0)	
2013–2014	218 (27.7)	0 (0.0)	
2015–2016	0 (0.0)	193 (100.0)	
Number of transplantations			0.16
1	672 (85.5)	173 (89.6)	
≥2	114 (14.5)	20 (10.4)	
Donor type			0.153
Related	109 (13.9)	19 (9.8)	
Unrelated	677 (86.1)	174 (90.2)	
HLA compatibility			0.12
Match	623 (79.3)	143 (74.1)	
Mismatch	163 (20.7)	50 (25.9)	
Conditioning regimen			0.001
CY+TBI	102 (13.0)	25 (13.0)	
BU+CY	122 (15.5)	21 (10.9)	
CA+CY+TBI	61 (7.8)	11 (5.7)	
FLU+(BU or MEL)	373 (47.5)	89 (46.1)	
FLU+(BU or MEL)+(BU or MEL or CA or CY)	66 (8.4)	37 (19.2)	
Other regimen	62 (7.9)	10 (5.2)	
GVHD prophylaxis			0.001
CSA+MTX	182 (23.2)	22 (11.4)	
TAC+MMF	12 (1.5)	5 (2.6)	
TAC+MTX	541 (68.8)	159 (82.4)	
CSA+MMF	5 (0.6)	0 (0.0)	
Other	46 (5.9)	7 (3.6)	
Use of ATG			0.005
No	750 (95.4)	173 (89.6)	
Yes	36 (4.6)	20 (10.4)	
Treatment for AML after transplantation			0.035
No	613 (78.0)	135 (69.9)	
Yes	170 (21.6)	58 (30.1)	
Missing	3 (0.4)	0 (0.0)	

Note

*p*‐values were calculated by Fisher's exact test or Wilcoxon Mann–Whitney test based on categorical or continuous variables.

Abbreviations: AML, acute myeloid leukemia; ATG, anti‐thymocyte globulin; BU, busulfan; CA, cytarabine; CR, complete remission; CSA, cyclosporine; CY, cyclophosphamide; FAB, French‐American‐British; FLU, fludarabine; GVHD, graft versus host disease; HCT‐CI, hematopoietic cell transplantation‐comorbidity index; IQR, interquartile range; MEL, melphalan; MMF, mycophenolate mofetil; MTX, methotrexate; TAC, tacrolimus; TBI, total body irradiation.

^a^ Continuous variable.

^b^ Cytogenetic risk determined by this study.

**TABLE 3:** Characteristics of patients who underwent peripheral blood stem cell transplantation

	Development cohort	Validation cohort	
	(*n* = 427); *n* (%) or median [IQR]	(*n* = 135); *n* (%) or median [IQR]	*p*
Age (years)^a^	50 [37, 59]	48 [37.5, 58]	0.762
Sex			0.612
Female	167 (39.1)	49 (36.3)	
Male	260 (60.9)	86 (63.7)	
ECOG performance status			0.115
0	118 (27.6)	50 (37.0)	
1	208 (48.7)	54 (40.0)	
2	69 (16.2)	17 (12.6)	
3	24 (5.6)	12 (8.9)	
4	8 (1.9)	2 (1.5)	
HCT‐CI			0.097
0	235 (55.0)	59 (43.7)	
1–3	154 (36.1)	64 (47.4)	
4–6	34 (8.0)	11 (8.1)	
≥7	4 (0.9)	1 (0.7)	
Peripheral blasts (%)^a^	6.00 [0.00, 30.0]	5.00 [0.00, 30.5]	0.847
FAB classification			0.29
M0	33 (7.7)	12 (8.9)	
M1–2	223 (52.2)	79 (58.5)	
M4–5	84 (19.7)	24 (17.8)	
M6	22 (5.2)	9 (6.7)	
M7	11 (2.6)	3 (2.2)	
Other	54 (12.6)	8 (5.9)	
Cytogenetic risk^b^			0.633
Favorable	243 (56.9)	79 (58.5)	
Intermediate	142 (33.3)	40 (29.6)	
Poor	42 (9.8)	16 (11.9)	
Response to chemotherapy			0.003
Primary induction failure	194 (45.4)	68 (50.4)	
Duration of first CR, <6 month	75 (17.6)	37 (27.4)	
Duration of first CR, ≥6 month	103 (24.1)	16 (11.9)	
No treatment before transplantation	55 (12.9)	14 (10.4)	
Disease status			1
De novo AML	383 (89.7)	122 (90.4)	
Secondary AML	44 (10.3)	13 (9.6)	
Year of transplantation			<0.001
2000–2010	157 (36.8)	0 (0.0)	
2011–2012	136 (31.9)	0 (0.0)	
2013–2014	134 (31.4)	0 (0.0)	
2015–2016	0 (0.0)	135 (100.0)	
Number of transplantations			0.447
1	351 (82.2)	107 (79.3)	
≥2	76 (17.8)	28 (20.7)	
Donor type			0.003
Related	415 (97.2)	122 (90.4)	
Unrelated	12 (2.8)	13 (9.6)	
HLA compatibility			0.736
Match	319 (74.7)	99 (73.3)	
Mismatch	108 (25.3)	36 (26.7)	
Conditioning regimen			0.002
CY+TBI	70 (16.4)	20 (14.8)	
BU+CY	55 (12.9)	17 (12.6)	
CA+CY+TBI	28 (6.6)	2 (1.5)	
FLU+(BU or MEL)	167 (39.1)	51 (37.8)	
FLU+(BU or MEL) +(BU or MEL or CA or CY)	59 (13.8)	37 (27.4)	
Other regimen	48 (11.2)	8 (5.9)	
GVHD prophylaxis			0.112
CSA+MTX	246 (57.6)	62 (45.9)	
TAC+MMF	16 (3.7)	6 (4.4)	
TAC+MTX	87 (20.4)	39 (28.9)	
CSA+MMF	9 (2.1)	5 (3.7)	
Other	69 (16.2)	23 (17.0)	
Use of ATG			0.001
No	387 (90.6)	107 (79.3)	
Yes	40 (9.4)	28 (20.7)	
Treatment for AML after transplantation			0.418
No	305 (71.4)	89 (65.9)	
Yes	119 (27.9)	45 (33.3)	
Missing	3 (0.7)	1 (0.7)	

Note

*p*‐values were calculated by Fisher's exact test or Wilcoxon Mann–Whitney test based on categorical or continuous variables.

Abbreviations: AML, acute myeloid leukemia; ATG, anti‐thymocyte globulin; BU, busulfan; CA, cytarabine; CR, complete remission; CSA, cyclosporine; CY, cyclophosphamide; FAB, French‐American‐British; FLU, fludarabine; GVHD, graft versus host disease; HCT‐CI, hematopoietic cell transplantation‐comorbidity index; IQR, interquartile range; MEL, melphalan; MMF, mycophenolate mofetil; MTX, methotrexate; TAC, tacrolimus; TBI, total body irradiation.

^a^ Continuous variable.

^b^ Cytogenetic risk determined by this study.

Recently, several studies, including prospective randomized studies or a meta‐analysis, have compared reduced‐intensity conditioning (RIC) and myeloablative conditioning (MAC). ^26^, ^27^, ^28^, ^29^ However, these studies did not show a statistically significant difference between RIC and MAC in terms of OS. On the other hand, an excellent survival benefit was reported in individual conditioning regimens.^19^, ^30^, ^31^, ^32^ Therefore, we stratified conditioning regimens before HCT into six categories based on the combination of chemotherapy drugs and total body irradiation (TBI) of ≥8 Gy, rather than categorizing them based on intensity. AML was categorized according to the FAB classification.^22^ Donor‐recipient HLA‐A, HLA‐B, and HLA‐DR compatibilities were determined. Patients with a 6/6 match at HLA‐A, ‐B and ‐DR were placed in the matched group and those with ≥1 mismatch were placed in the mismatched group. We classified cytogenetic risk according to the National Comprehensive Cancer Network (NCCN) Guidelines Version 1.2016^33^ as favorable risk [t(8;21), t(15;17), inv(16), t(16;16)], poor risk [−5/del(5q), −7/del(7q), inv(3), t(3;3), 11q23 other than t(9;11), t(6;9), t(9;22), complex karyotype (CK), monosomal karyotype], or intermediate risk [normal, +8 alone, t(9;11), other non‐defined]. However, patients with favorable risk did not have a better prognosis than those with intermediate risk in that situation. We then revised the classification of cytogenetic risk based on the findings of univariable analysis (see Results).

### 2.3 Statistical analyses

OS was defined as the time from HCT to last contact or death from any cause. The OS rates were determined using the Kaplan–Meier method and analyzed using the log‐rank test. We used a Cox proportional hazards model for multivariate analysis; the prognostic factors from potential predictors were identified by applying backward stepwise selection and retaining the variables with *p* values <0.05. Nomograms and a web application were developed based on the results of the multivariate analyses. The accuracy of the prognostic models was validated through calibration (assessed by plotting the predicted vs. observed OS rates), discrimination (assessed by concordance probability estimate; c‐index^34^), and survival curves. A c‐index of 1 indicated perfect discrimination, while a c‐index of 0.5 indicated no discrimination. Internal validation of each prognostic model was performed using the bootstrap method with 1000 resamples for calibration and discrimination using the respective development cohorts. To validate each prognostic model, we used the respective validation cohort. Moreover, we applied a previously reported scoring system for patients with AML relapse or primary induction failure who underwent BMT and PBSCT^18^ to our validation cohort (cases with missing values were excluded). Briefly, the scoring system was based on the response to chemotherapy, cytogenetics, HLA‐match, circulating blasts, and Karnofsky score. Subsequently, the patients were categorized into four groups (scores of 0, 1, 2, and ≥3).

Analyses were performed using SAS (version 9.4, SAS Institute), SPSS (IBM Corp. Released 2011. IBM SPSS Statistics for Windows, Version 20.0. Armonk, NY: IBM Corp.), EZR,^35^ and R 3.2.3 software (https://www.r‐project.org) with package rms version 5.1–1 (https://cran.r‐project.org/web/packages/rms). The web application was developed using R 3.2.3 software with shiny version 1.0.5 (https://shiny.rstudio.com).

## 3 RESULTS

### 3.1 Characteristics and survival of patients

The characteristics of the patients in the development (CBT, *n* = 1077; BMT, *n* = 786; and PBSCT, *n* = 427) and validation cohorts (CBT, *n* = 434; BMT, *n* = 193; and PBSCT, *n* = 135) are listed in Tables 1, 2, 3. In the cohort, CBT was performed with a single unit, most bone marrow grafts were unrelated, and most peripheral blood grafts were related. The 1‐ and 5‐year OS rates in the development cohort were 31.1% (95% confidence interval [CI], 28.3%–34.0%) and 20.3% (95% CI, 17.5%–23.2%), respectively, after CBT; 37.2% (95% CI, 33.7%–40.6%) and 23.1% (95% CI, 19.7%–26.6%), respectively, after BMT; and 38.1% (95% CI, 33.3%–42.8%) and 18.9% (95% CI, 14.6%–23.6%), respectively, after PBSCT. The 1‐year OS rates in the validation cohort was 39.4% (95% CI, 34.5%–44.3%) after CBT, 33.8% (95% CI, 26.6%–41.2%) after BMT, and 37.4% (95% CI, 28.4%–46.4%) after PBSCT.

### 3.2 Identification of cytogenetic risk for allogenic hematopoietic stem cell transplantation in acute myeloid leukemia in non‐complete remission

The Kaplan–Meier curve was plotted based on the cytogenetic risk classified by the NCCN Guidelines (Figure S1A). However, patients with favorable risk did not have a better prognosis than those with intermediate risk. To identify the cytogenetic risk for allo‐HCT in non‐CR AML, we performed univariable analysis. Based on these results, the cytogenetic risk was classified as poor [−5/del(5q), −17, t(6;9), not evaluable], intermediate [CK, −7/del(7q), inv(3), t(3;3), 11q23 other than t(9;11), t(8;21)], or favorable [normal, inv(16), +8 alone, t(9;11), other non‐defined] (Table S1). If cytogenetic risk was categorized into two groups, the worse risk classification was adopted. This grouping successfully stratified patients with non‐CR AML who underwent allo‐HCT (Figure S1B).

### 3.3 Conditioning regimen of ≥3 drugs including fludarabine in cord blood transplantation was associated with favorable overall survival and leukemia‐free survival

**FIGURE 2:**
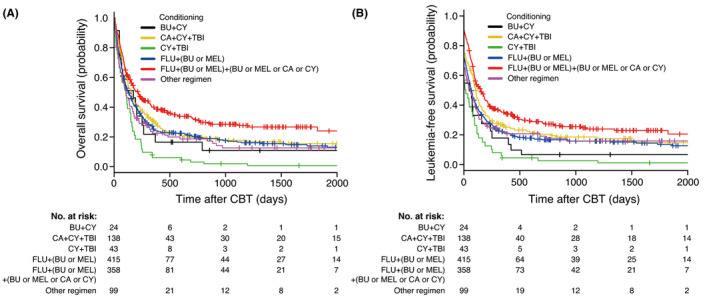
Overall survival and leukemia‐free survival after cord blood transplantation. The estimates for the overall survival (A) and leukemia‐free survival (B) of patients in the development cohort were adjusted for the age at transplantation, sex, performance status, hematopoietic cell transplantation‐comorbidity index, percentage of peripheral blasts, cytogenetic risk classification, response to chemotherapy, and number of transplantations. The hazard ratio for the overall survival of patients in the FLU+(BU or MEL) group versus those in the FLU + (BU or MEL) + (BU, MEL, CA, or CY) group was 1.442 (95% confidence interval [CI], 1.211–1.718; *p* < 0.0001); in the CA +CY + TBI group versus those in the FLU + (BU or MEL) + (BU, MEL, CA, or CY) group was 1.303 (95% CI, 0.992–1.712; *p* = 0.0574); in the BU +CY group versus those in the FLU + (BU or MEL) + (BU, MEL, CA, or CY) group was 1.576 (95% CI, 0.966–2.571; *p* = 0.0685). The hazard ratio for the leukemia‐free survival of patients in the FLU + (BU or MEL) group versus those in the FLU + (BU or MEL) + (BU, MEL, CA, or CY) group was 1.533 (95% CI, 1.294–1.817; *p* < 0.0001); in the CA +CY + TBI group versus those in the FLU + (BU or MEL) + (BU, MEL, CA, or CY) group was 1.320 (95% CI, 1.013–1.719; *p* = 0.0395); in the BU +CY group versus those in the FLU + (BU or MEL) + (BU, MEL, or CA, or CY) group was 2.045 (95% CI, 1.282–3.263; *p* = 0.0027). BU, busulfan; CA, cytarabine; CBT, cord blood transplantation; CY, cyclophosphamide; FLU, fludarabine; MEL, melphalan; TBI, total body irradiation

Using the backward stepwise selection method in the Cox proportional hazards model, we identified the following significant prognostic factors for OS in patients in the development cohort who underwent CBT: age of the recipient at transplantation, sex, ECOG PS, HCT‐CI, percentage of peripheral blasts, cytogenetic risk classification, response to chemotherapy, number of transplantations, and conditioning regimen (Table 4). Interestingly, compared with cyclophosphamide/TBI (conditioning regimen), the use of ≥3 drugs (including fludarabine) with CBT showed the lowest hazard ratio for mortality (0.384; 95% CI, 0.266–0.554; *p *< 0.0001). Among all conditioning regimens, the use of ≥3 drugs (including fludarabine) with CBT showed the best leukemia‐free survival (LFS) and favorable OS (Figure 2), whereas the regimen with BMT or PBSCT did not show the best prognosis (Figure S2). Table S2 lists the details of the ≥3 drug regimen, including fludarabine, administered with CBT. A combination of fludarabine, melphalan, and busulfan (FLU/BU/MEL) was most frequently used (34.9%). Similar to those in patients undergoing CBT, the age of the recipient at transplantation, ECOG PS, HCT‐CI, percentage of peripheral blasts, FAB classification, cytogenetic risk classification, response to chemotherapy, and number of transplantations were identified as significant prognostic factors for OS in patients who underwent BMT (Table 5); age of the recipient at transplantation, sex, ECOG PS, percentage of peripheral blasts, cytogenetic risk classification, response to chemotherapy, and number of transplantations were also identified as significant prognostic factors for OS in patients who underwent PBSCT (Table 6). The conditioning regimen was a significant prognostic factor for OS in only patients who underwent CBT; the common significant prognostic factors among the three types of HCTs were age of the recipient at transplantation, ECOG PS, percentage of peripheral blasts, cytogenetic risk classification, response to chemotherapy, and number of transplantations.

**TABLE 4:** Results of the multivariate analysis of the overall survival of patients who underwent cord blood transplantation

	HR	95% CI	*p*
Age (per year)^a^	1.014	1.008–1.02	<0.0001
Sex			
Female	1.000		
Male	1.404	1.210–1.628	<0.0001
ECOG performance status			
0	1.000		
1	1.310	1.080–1.588	0.0061
2	1.801	1.429–2.269	<0.0001
3	3.386	2.538–4.519	<0.0001
4	7.703	4.898–12.116	<0.0001
HCT‐CI			
0	1.000		
1–3	1.133	0.967–1.328	0.1211
4–6	1.215	0.958–1.543	0.1089
≥7	2.060	1.221–3.475	0.0068
Peripheral blasts (per percentage)^a^	1.005	1.003–1.007	<0.0001
Cytogenetic risk^b^			
Favorable	1.000		
Intermediate	1.316	1.124–1.54	0.0006
Poor	1.596	1.286–1.98	<0.0001
Response to chemotherapy			
Primary induction failure	1.000		
Duration of first CR, <6 month	1.328	1.087–1.622	0.0056
Duration of first CR, ≥6 month	0.971	0.799–1.18	0.7666
No treatment before transplantation	0.713	0.566–0.899	0.0042
Number of transplantations			
1	1.000		
≥2	1.589	1.312–1.924	<0.0001
Conditioning regimen			
CY+TBI	1.000		
BU+CY	0.605	0.343–1.067	0.0828
CA+CY+TBI	0.501	0.340–0.737	0.0005
FLU+(BU or MEL)	0.554	0.387–0.792	0.0012
FLU+(BU or MEL)+(BU, MEL, CA, or CY)	0.384	0.266–0.554	<0.0001
Other regimen	0.624	0.416–0.936	0.0227

Abbreviations: BU, busulfan; CA, cytarabine; CR, complete remission; CY, cyclophosphamide; FLU, fludarabine; HCT‐CI, hematopoietic cell transplantation‐comorbidity index; HR, hazard ratio; MEL, melphalan; TBI, total body irradiation.

^a^ Continuous variable.

^b^ Cytogenetic risk determined by this study.

**TABLE 5:** Results of the multivariable analysis of the overall survival of patients who underwent bone marrow transplantation

	HR	95% CI	*p*
Age (per year)^a^	1.012	1.005–1.019	0.001
ECOG performance status			
0	1.000		
1	1.078	0.883–1.317	0.458
2	1.870	1.441–2.429	<0.0001
3	1.887	1.273–2.797	0.0016
4	6.445	3.301–12.583	<0.0001
HCT‐CI			
0	1.000		
1–3	1.223	1.020–1.466	0.03
4–6	1.954	1.451–2.633	<0.0001
≥7	3.352	1.707–6.584	0.0004
Peripheral blasts (per percentage)^a^	1.010	1.007–1.013	<0.0001
FAB classification			
M0	1.000		
M1–2	1.090	0.804–1.476	0.5795
M4–5	1.168	0.835–1.633	0.3651
M6	1.374	0.926–2.038	0.1146
M7	1.980	1.113–3.522	0.0201
Other	0.809	0.546–1.196	0.2875
Cytogenetic risk^b^			
Favorable	1.000		
Intermediate	1.407	1.166–1.697	0.0004
Poor	1.899	1.473–2.449	<0.0001
Response to chemotherapy			
Primary induction failure	1.000		
Duration of first CR, <6 month	1.235	0.996–1.531	0.0549
Duration of first CR, ≥6 month	0.781	0.614–0.994	0.0446
No treatment before transplantation	0.703	0.515–0.960	0.0267
Number of transplantations			
1	1.000		
≥2	1.359	1.058–1.747	0.0164

Abbreviations: CR, complete remission; FAB, French‐American‐British; HCT‐CI, hematopoietic cell transplantation‐comorbidity index; HR, hazard ratio.

^a^ Continuous variable.

^b^ Cytogenetic risk determined by this study.

**TABLE 6:** Results of the multivariable analysis of the overall survival of patients who underwent peripheral blood stem cell transplantation

	HR	95% CI	*p*
Age (per year)^a^	1.023	1.014–1.032	<0.0001
Sex			
Female	1.000		
Male	1.347	1.061–1.711	0.0146
ECOG performance status			
0	1.000		
1	1.213	0.912–1.614	0.1848
2	1.464	1.017–2.106	0.0402
3	2.862	1.706–4.800	<0.0001
4	4.750	2.120–10.643	0.0002
Peripheral blasts (%)^a^	1.009	1.004–1.013	<0.0001
Cytogenetic risk^b^			
Favorable	1.000		
Intermediate	1.438	1.117–1.851	0.0048
Poor	1.592	1.089–2.328	0.0164
Response to chemotherapy			
Primary induction failure	1.000		
Duration of first CR, <6 month	1.458	1.054–2.017	0.0229
Duration of first CR, ≥6 month	1.234	0.907–1.678	0.1808
No treatment before transplantation	0.532	0.355–0.798	0.0023
Number of transplantations			
1	1.000		
≥2	1.402	1.008–1.949	0.0447

Abbreviations: CR, complete remission; HR, hazard ratio.

^a^ Continuous variable.

^b^ Cytogenetic risk determined by this study.

### 3.4 Development and validation of nomograms

**FIGURE 3:**
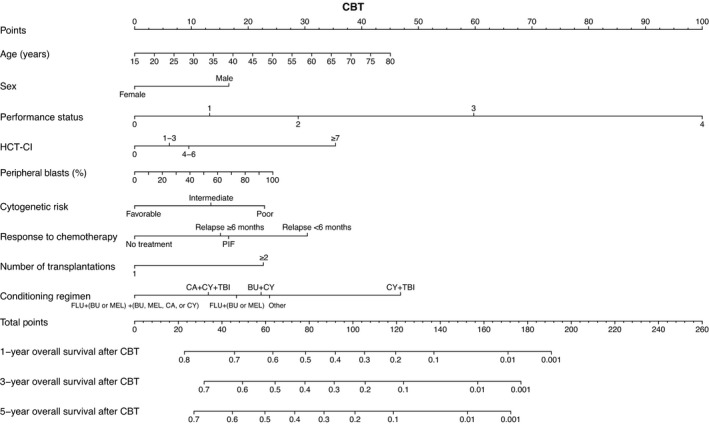
Nomogram to predict the overall survival after cord blood transplantation. This nomogram predicts the 1‐, 3‐, and 5‐year overall survival probabilities of patients with acute myeloid leukemia undergoing cord blood transplantation in non‐complete remission. BU, busulfan; CA, cytarabine; CBT, cord blood transplantation; CY, cyclophosphamide; FLU, fludarabine; HCT‐CI, hematopoietic cell transplantation comorbidity index; MEL, melphalan; PIF, primary induction failure; Relapse ≥6 months, the duration of the first complete remission was ≥6 months; Relapse <6 months, the duration of the first complete remission was <6 months; TBI, total body irradiation

**FIGURE 4:**
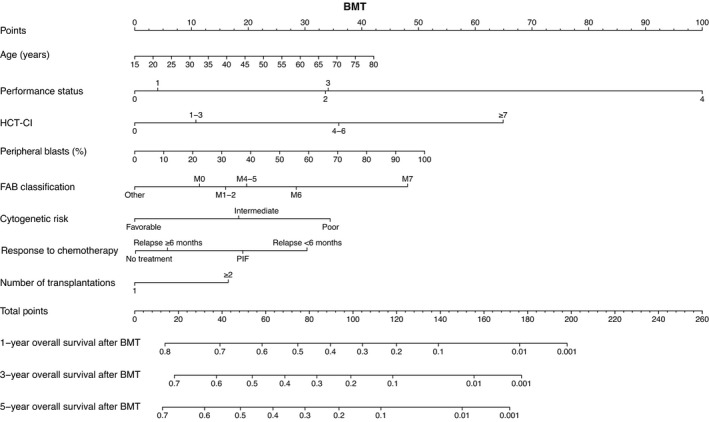
Nomogram to predict overall survival after bone marrow transplantation. This nomogram predicts the 1‐, 3‐, and 5‐year overall survival probabilities of patients with acute myeloid leukemia undergoing bone marrow transplantation in non‐complete remission. BMT, bone marrow transplantation; FAB, French‐American‐British; HCT‐CI, hematopoietic cell transplantation comorbidity index; PIF, primary induction failure; Relapse ≥6 months, the duration of the first complete remission was ≥6 months; Relapse <6 months, the duration of the first complete remission was <6 months

**FIGURE 5:**
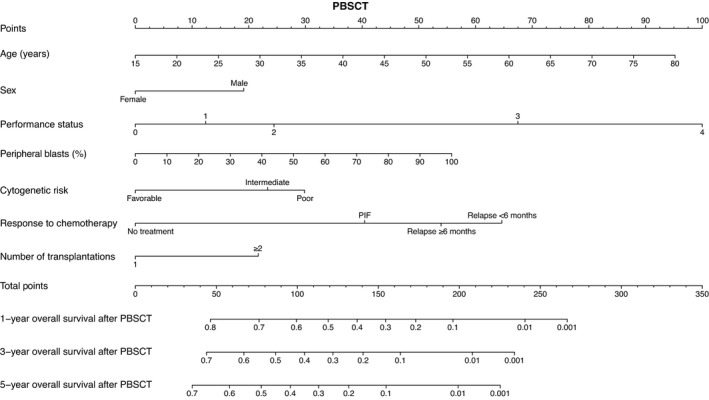
Nomogram to predict overall survival after peripheral blood stem cell transplantation. This nomogram predicts the 1‐, 3‐, and 5‐year overall survival probabilities of patients with acute myeloid leukemia undergoing peripheral blood stem cell transplantation in non‐complete remission. PBSCT, peripheral blood stem cell transplantation; PIF, primary induction failure; Relapse ≥6 months, the duration of the first complete remission was ≥6 months; Relapse <6 months, the duration of the first complete remission was <6 months

**FIGURE 6:**
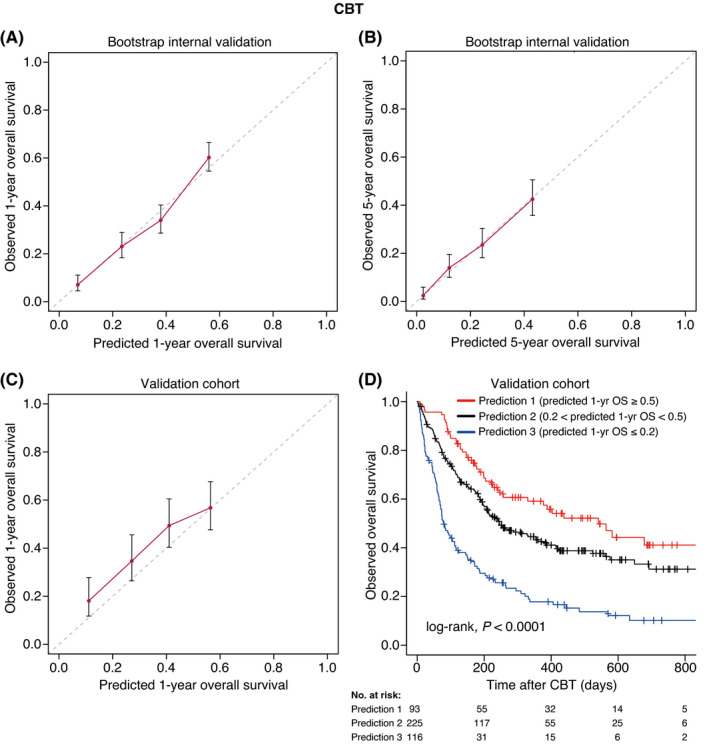
Validation of the overall survival nomogram for cord blood transplantation. In the upper panels, the calibration plots show the bootstrap internal validation for the 1‐ (A) and 5‐year (B) overall survival. The lower left panel shows the calibration plot of 1‐year overall survival in the validation cohort (C). The x‐axis represents the overall survival rate predicted by the nomogram. The y‐axis represents the observed overall survival rate estimated using the Kaplan–Meier method. Patients were divided into four groups of equal size based on the predicted overall survival rate. The dashed line shows the ideal line, which indicates that the predicted overall survival rate is the same as the observed overall survival rate. The dots show the median values, and error bars show 95% CIs. Kaplan–Meier curves according to nomogram predictions are shown (D). CBT indicates cord blood transplantation; 1‐yr, 1‐year; OS, overall survival

**FIGURE 7:**
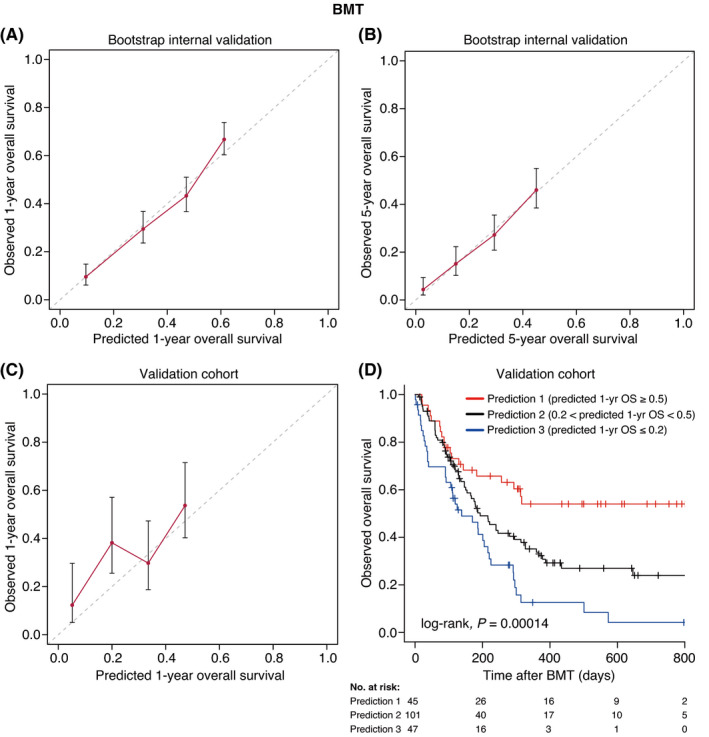
Validation of the overall survival nomogram for bone marrow transplantation. In upper panels, the calibration plots show the bootstrap internal validation for the 1‐ (A) and 5‐year (B) overall survival. The lower left panel shows the calibration plot of 1‐year overall survival in the validation cohort (C). The x‐axis represents the overall survival rate predicted by the nomogram. The y‐axis represents the observed overall survival rate estimated by the Kaplan–Meier method. Patients were divided into four groups of equal size based on the predicted overall survival rate. The dashed line shows the ideal line, which represents that the predicted overall survival rate is the same as the observed overall survival rate. The dots show the median values and error bars show 95% CIs. The Kaplan‐Meier curves according to prediction by the nomogram are shown (D). BMT, bone marrow transplantation; 1‐yr, 1‐year; OS, overall survival

**FIGURE 8:**
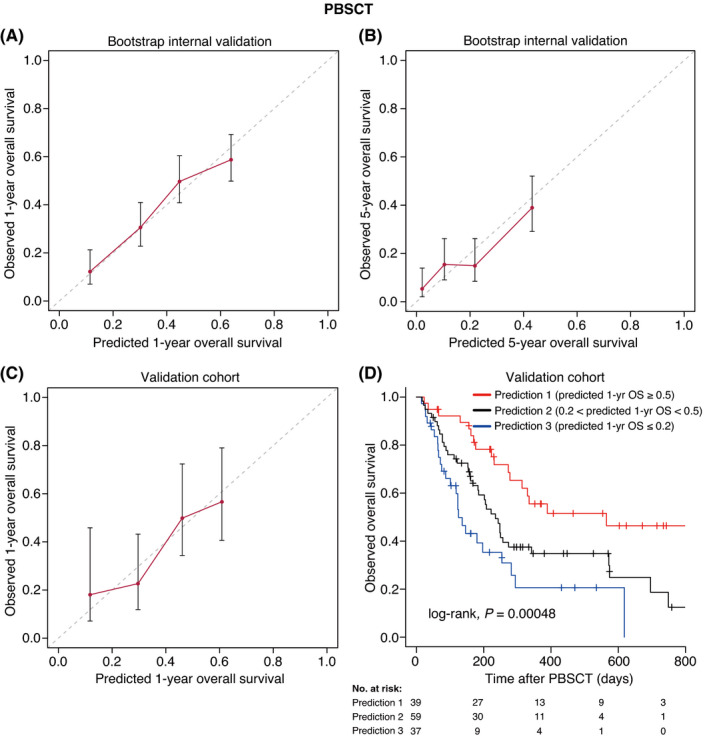
Validation of the overall survival nomogram for peripheral blood stem cell transplantation. In upper panels, the calibration plots show the bootstrap internal validation for the 1‐ (A) and 5‐year (B) overall survival. The lower left panel shows the calibration plot of 1‐year overall survival in the validation cohort (C). The x‐axis represents the overall survival rate predicted by the nomogram. The y‐axis represents the observed overall survival rate estimated by the Kaplan–Meier method. Patients were divided into four groups of equal size based on the predicted overall survival rate. The dashed line shows the ideal line, which represents that the predicted overall survival rate is the same as the observed overall survival rate. The dots show the median values and error bars show 95% CIs. The Kaplan–Meier curves according to prediction by the nomogram are shown (D). PBSCT, peripheral blood stem cell transplantation; 1‐yr, 1‐year; OS, overall survival

**FIGURE 9:**
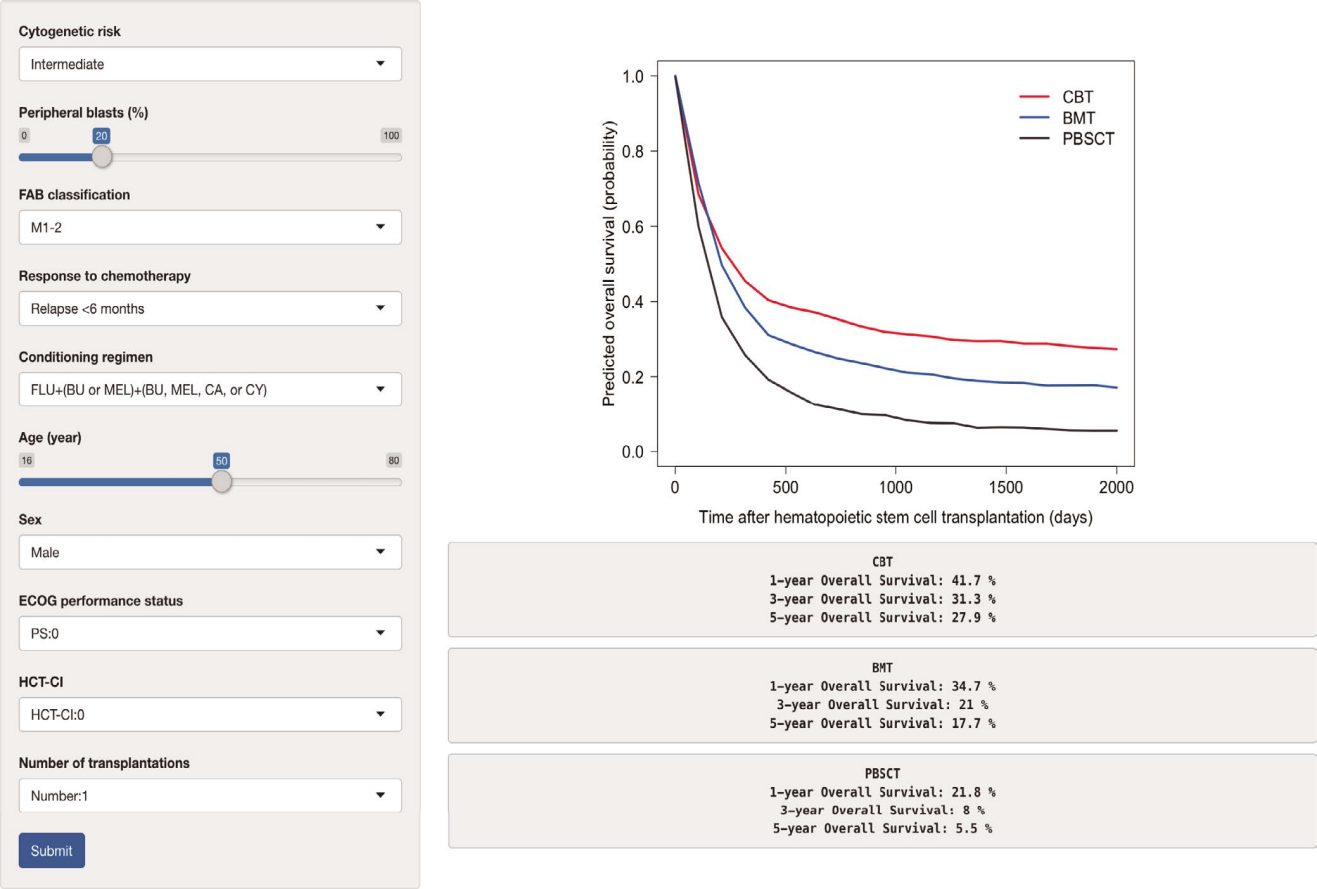
Web application to predict the overall survival following three types of transplantations. The web application is available at https://JSHCT‐AMLWG.shinyapps.io/Predict‐OS‐nonCR‐AML‐post‐HCT/. BMT, bone marrow transplantation; BU, busulfan; CA, cytarabine; CBT, cord blood transplantation; CY, cyclophosphamide; FLU, fludarabine; HCT‐CI, hematopoietic cell transplantation‐comorbidity index; FAB, French‐American‐British; MEL, melphalan; PBSCT, peripheral blood stem cell transplantation; TBI, total body irradiation

Based on the results of the multivariate analyses, we constructed nomograms to predict the 1‐, 3‐, and 5‐year OS of patients after CBT, BMT, and PBSCT (Figures 3, 4, 5). The point of each characteristic was determined by drawing an upward vertical line from the covariate to the points axis. The total points score was obtained by summing each point. The 1‐, 3‐, and 5‐year overall survival probabilities were determined by drawing a downward vertical line from the total points score. Next, we validated the performance of the prognostic models. Figures 6A and B, 7A and B and 8A and B show the calibration plots of the 1‐ and 5‐year OS for CBT, BMT and PBSCT in the development cohort using the bootstrap method, and Figures 6C, 7C and 8C show the calibration plot of 1‐year OS in the validation cohort. Sample points lie on the diagonal line when the predicted OS is equal to the observed OS. The calibration plots correlated well with the predicted and observed OS, indicating the accuracy of the prognostic models. Furthermore, we confirmed that the actual Kaplan–Meier curves in the validation cohort were successfully stratified by our nomograms (Figures 6D, 7D and 8D). In the internal validation, the bootstrap‐corrected c‐indices of the nomograms for CBT, BMT, and PBSCT were 0.671 (95% CI, 0.652–0.690), 0.675 (95% CI, 0.652–0.699), and 0.654 (95% CI, 0.621–0.688), respectively. In the validation cohort, the c‐indices of the nomograms for CBT, BMT, and PBSCT were 0.648 (95% CI, 0.613–0.682), 0.600 (95% CI, 0.542–0.658), and 0.658 (95% CI, 0.596–0.720), respectively. Using a previous scoring system,^18^ the c‐indices for BMT and PBSCT were 0.587 (95% CI, 0.529–0.645) and 0.570 (95% CI, 0.491–0.650), respectively. The distribution of scores in the validation cohort is given in Table S3. These data indicate that our nomograms were at least as accurate as the previous scoring system. We also developed a web application (https://JSHCT‐AMLWG.shinyapps.io/Predict‐OS‐non‐CR‐AML‐post‐HCT/) based on these prognostic models. This enabled us to simultaneously estimate the prognosis and construct survival curves after CBT, BMT, and PBSCT with ease (Figure 9).

## 4 DISCUSSION

We developed three nomograms and a web application to predict the 1‐, 3‐, and 5‐year OS of patients with AML in non‐CR after CBT, BMT, and PBSCT. We validated the nomograms showing adequate calibration and discrimination despite the diversity in patient characteristics, leukemia subtype, and treatments.

In this study, we revealed the common significant prognostic factors for the three types of HCTs. These factors were attributed to patient characteristics and tumor characteristics and not to treatment. Intriguingly, the conditioning regimen that physicians selected was a significant prognostic factor only in CBT. A previous single‐arm study showed excellent survival outcomes (2‐year OS rate =54.9%; 2‐year progression‐free survival rate =54.9%) of patients with myeloid malignancies in non‐CR who underwent CBT and were treated with FLU/BU/MEL.^32^ Notably, we demonstrated that the use of a ≥3 drug regimen, including fludarabine, such as the combination FLU/BU/MEL, resulted in a favorable prognosis, but the conditioning regimen was not a significant prognostic factor for the OS of patients undergoing BMT or PBSCT. It was reported that cyclophosphamide/TBI supplemented with high‐dose cytarabine was effective for patients undergoing CBT but not for those undergoing BMT or PBSCT,^19^, ^36^ which is in accordance with the findings of our study. The distinct difference may be due to differences in the composition and properties of cord blood and bone marrow or peripheral blood.^37^ Our data suggests the importance of selecting appropriate conditioning regimens for each donor source.

The ≥3 drug regimen such as FLU/BU/MEL had a positive impact on prognosis. This is because the respective chemotherapy drugs may have different anti‐tumor mechanisms. For example, fludarabine inhibits DNA/RNA synthesis by incorporating the drug into DNA or RNA.^38^, ^39^ Melphalan and busulfan are alkylating agents, but melphalan is classified as nitrogen mustards and busulfan as alkyl alkane sulfonates.^40^ Melphalan reacts with N7‐guanine, N3‐adenine, and O6‐guanine in DNA to form covalent alkyl lesions.^41^ Whereas, busulfan reacts with not only N7‐guanine and N3‐adenine in DNA, but also with proteins.^40^ Furthermore, busulfan does not elicit toxicity via alkylation of O6‐guanine.^42^ Thus, the combination of drugs with different mechanisms may be useful in enhancing the anti‐tumor effect and eradicating leukemia cells. Actually, a previous study showed that fludarabine and double alkylating agents (busulfan and thiotepa) could enhance the anti‐tumor effect compared with fludarabine and a single‐alkylating agent (busulfan).^30^

It was previously reported that circulating blasts, cytogenetic risk, duration of first CR, and Karnofsky or Lansky score significantly affected the OS of patients with relapsed AML or failure in primary induction who underwent BMT or PBSCT.^18^ In our study, they were also selected as prognostic factors for CBT as well as for BMT and PBSCT. Moreover, we found that an increase in the number of transplantations was associated with a poor prognosis for any stem cell source. This might be attributed to the condition of patients with AML and an increase in leukemic stem cell frequency and heterogeneity after unsuccessful treatment.^24^, ^25^

Commonly used risk scores to predict the OS of patients with AML in relapse or with primary induction failure undergoing BMT and PBSCT have been developed using a large cohort.^18^ In the commonly used risk scores, each prognostic factor has an equal prognostic weight in the outcome despite having a different hazard ratio, which results in a reduction of the predictive accuracy of the prognostic model.^43^ However, each hazard ratio in this study was accurately represented in the prognostic model. Various studies have documented the superiority of the method used in this study over risk categorization.^43^, ^44^ We selected candidate predictors that they have not been previously included, such as HCT‐CI, FAB classification, and number of transplantations. Moreover, the model included data from pediatric AML patients; however, recent studies have indicated a distinct difference in biological and molecular profiling between pediatric and adult AML.^45^, ^46^ Therefore, to develop a prognostic model suitable for adult AML patients, we focused only on data from adult patients. These reasons could have resulted in the improved performance of our prognostic models compared with that of the previous scoring system.^18^ Furthermore, our prognostic models can compare the prognosis of different types of transplantations. They can be useful because there have been no randomized trials to determine appropriate donor sources.^47^

Recently, the use of haploidentical transplantation has been increasing for refractory AML. However, there are a few retrospective studies comparing haploidentical transplantation with other transplants for refractory AML, and there are no published randomized clinical trials. Suitable situations for haploidentical transplantation are not yet fully understood. It was reported that haploidentical transplantation for refractory/relapsed AML was associated with shorter GVHD‐free relapse‐free survival, inferior LFS, and shorter OS than transplantation from an HLA‐identical sibling, mainly due to infections,^48^ whereas another report showed no differences in GVHD‐free relapse‐free survival, LFS, or OS between haploidentical transplants and transplants from HLA‐identical siblings for AML in first CR with high‐risk cytogenetics.^49^ As our data could be used to estimate OS adjusted for the characteristics of patients after allo‐HCTs, except for haploidentical transplantation, it may be useful for a reference when haploidentical results are evaluated.

It is important to note the limitations of this study. First, the regimens for haploidentical transplantation were heterogeneous in our cohort because of limited previous evidence, and the number of transplantations was insufficient to build an accurate prognostic model. Therefore, haploidentical transplantation was excluded. Second, in this study, we used a Japanese cohort, which differs from other populations in some aspects. For example, in the US, most CBTs in adults are performed with double‐unit cord blood grafts, whereas in Japan, CBTs in adults are performed with a single unit. Moreover, for unrelated transplantations, in the US, most grafts are derived from peripheral blood, whereas in Japan, most grafts are derived from bone marrow. Such differences may limit the generalizability of the findings and prognostic models. Therefore, our findings must be validated using data from other countries. Third, comprehensive genomic studies on AML using next‐generation sequencing have recently revealed the relevance of clinical outcomes.^23^, ^50^, ^51^ However, data on somatic mutations were not available. Thus, in future studies, genomic information should be incorporated for developing effective prognostic models.

In conclusion, we designed and validated novel nomograms and a web application to predict the OS of patients with AML undergoing allo‐HCTs in non‐CR, indicating that the performance of our models was at least as favorable as that of the previous scoring system. These prognostic models can be helpful in estimating the benefits and risks of a patient and can provide clues as to whether to conduct transplantation when encountering a patient with AML in non‐CR. Furthermore, the web application enables us to easily compare the OS in a variety of settings; therefore, the study can be useful for designing prospective clinical trials. Moreover, our study revealed that the use of multiple chemotherapeutic drugs in CBT greatly contributed to the prognosis of patients with non‐CR AML.

## CONFLICT OF INTEREST

The authors have no conflict of interest associated with this work.

## ETHICAL STATEMENT

This study complied with the ethical principles of the Declaration of Helsinki and was approved by the data management committees of TRUMP and the Ethics Committee of Kyoto University, where this study was conducted. Written informed consent was obtained from all patients.

## Supporting information

Fig S1‐S2

Table S1

Table S2

Table S3

## Data Availability

The data were obtained from the TRUMP database and are not publicly available.
