# Supplementary material for: Personalized prediction of overall survival in patients with AML in non‐complete remission undergoing allo‐HCT
Source: Cancer Med. 2021 Jun 16;10(13):4250–68. doi: 10.1002/cam4.3920 (PMC8267144; doi:10.1002/cam4.3920)
Supplement: Supplementary file 1 — Fig S1‐S2 [file CAM4-10-4250-s001.pdf]

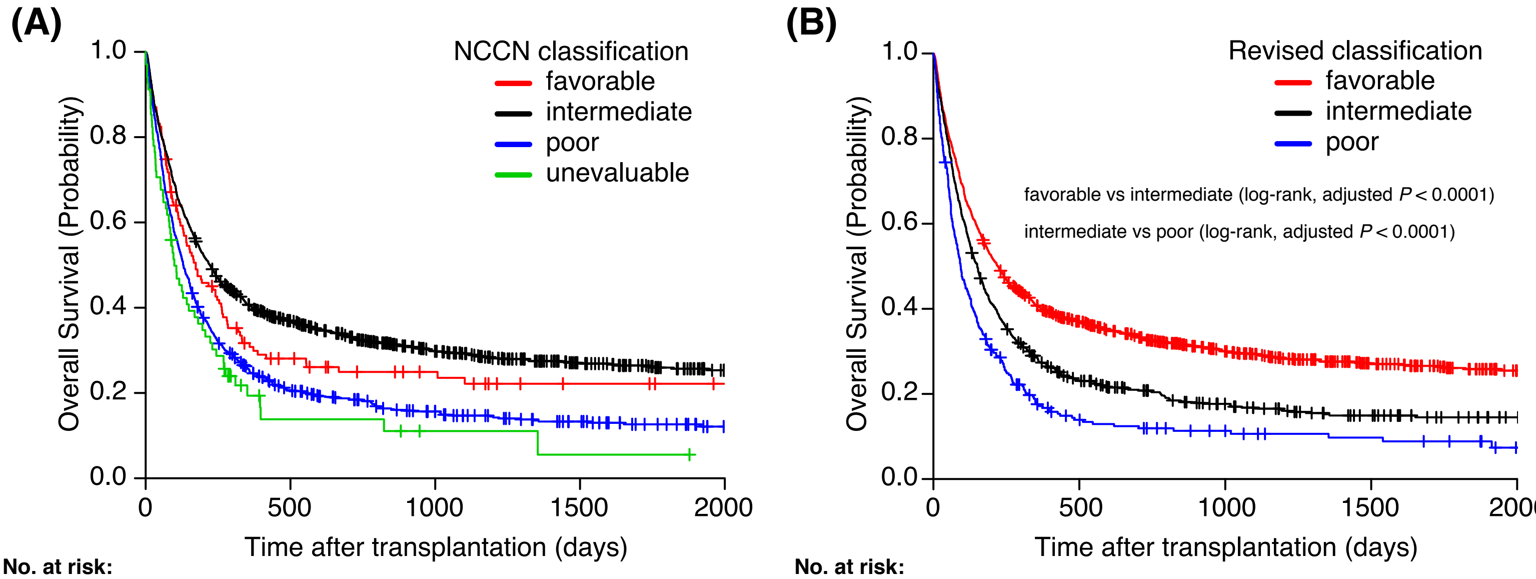


**No. at risk:**

| | 0 | 500 | 1000 | 1500 | 2000 |
|---|---|---|---|---|---|
| favorable | 132 | 29 | 18 | 10 | 7 |
| intermediate | 1412 | 403 | 234 | 134 | 68 |
| poor | 922 | 148 | 82 | 48 | 19 |
| unevaluable | 68 | 5 | 2 | 1 | 0 |

**No. at risk:**

| | 0 | 500 | 1000 | 1500 | 2000 |
|---|---|---|---|---|---|
| favorable | 1434 | 411 | 239 | 136 | 69 |
| intermediate | 803 | 145 | 80 | 46 | 22 |
| poor | 297 | 29 | 17 | 11 | 3 |

**FIGURE S1. Unadjusted estimates of overall survival based on cytogenetic risk classification.**

(A) The Kaplan–Meier plot shows the overall survival of patients in the development cohort according to the NCCN cytogenetic classification.

(B) The Kaplan–Meier plot shows the overall survival of the patients in the development cohort according to the revised cytogenetic risk classification (also see Table S1). The hazard ratio for the overall survival in the intermediate group versus that in the favorable group was 1.387 (95% CI, 1.255-1.533), and the hazard ratio for overall survival in the poor group versus that in the intermediate group was 1.364 (95% CI, 1.179-1.578).

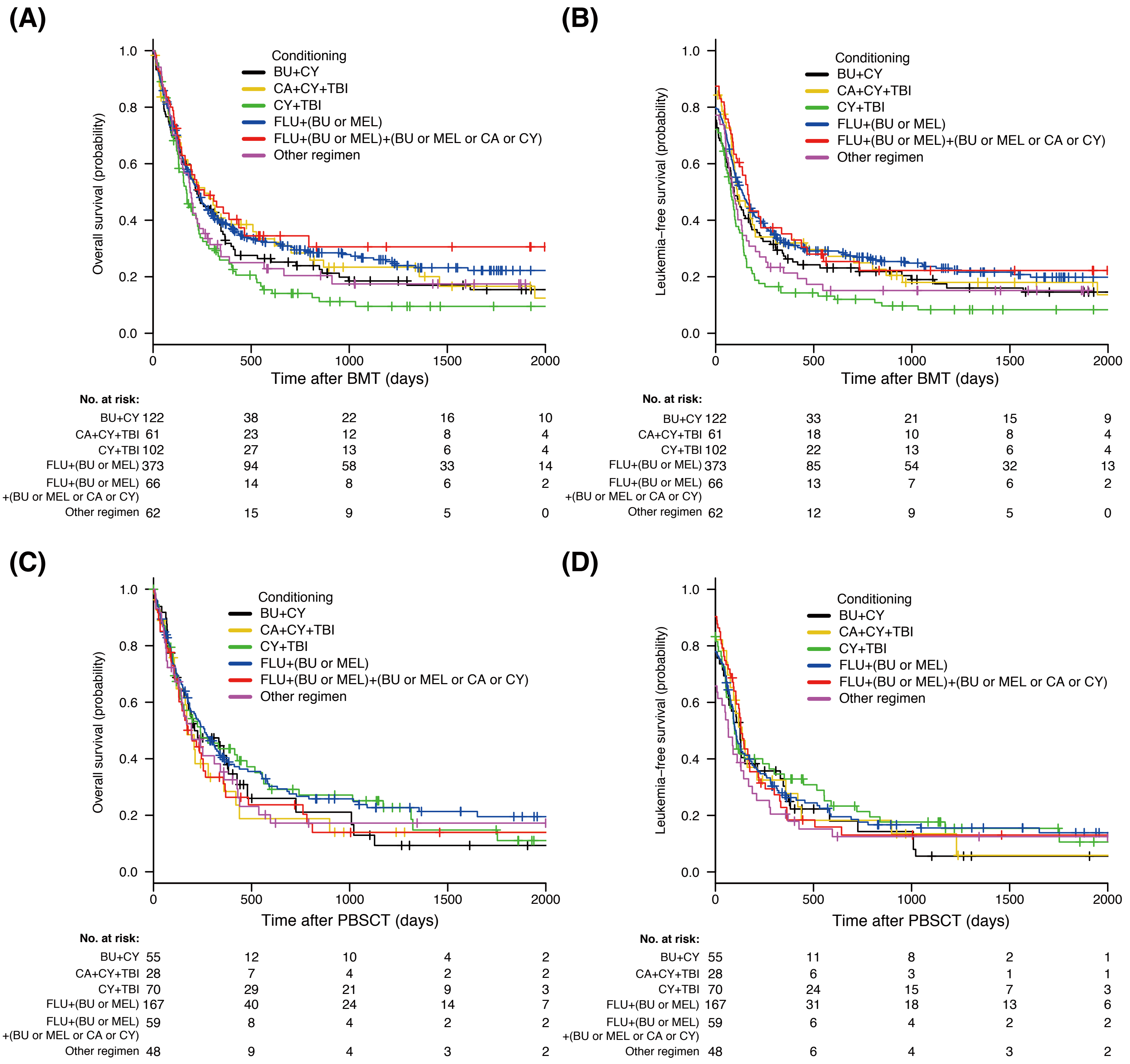


**FIGURE S2. Adjusted estimates of overall survival and leukemia-free survival after bone marrow transplantation and peripheral blood stem cell transplantation.**

The estimates for the overall survival (A) and leukemia-free survival (B) of patients in the development cohort who underwent bone marrow transplantation (n = 786) were adjusted for the age at transplantation, performance status, hematopoietic cell transplantation-comorbidity index, percentage of peripheral blasts, French-American-British classification, cytogenetic risk classification, response to chemotherapy, and times of transplantation. The survival curves were stratified according to the conditioning regimens. The hazard ratio for the overall survival of patients in the FLU+(BU or MEL) group versus those in the FLU+(BU or MEL)+(BU or MEL or CA or CY) group was 1.135 (95% CI, 0.829-1.553; P = 0.429); in the CA+CY+TBI group versus those in the FLU+(BU or MEL)+(BU or MEL or CA or CY) group was 1.187 (95% CI, 0.760-1.855; P = 0.451); in the BU+CY group versus those in the FLU+(BU or MEL)+(BU or MEL or CA or CY) group was 1.274 (95% CI, 0.877-1.850; P = 0.204). The hazard ratio for leukemia-free survival of patients in the FLU+(BU or MEL) group versus those in the FLU+(BU or MEL)+(BU or MEL or CA or CY) group was 1.113 (95% CI, 0.823-1.505; P = 0.488); in the CA+CY+TBI group versus those in the FLU+(BU or MEL)+(BU or MEL or CA or CY) group was 1.141 (95% CI, 0.741-1.758; P = 0.548); in the BU+CY group versus those in the FLU+(BU or MEL)+(BU or MEL or CA or CY) group was 1.309 (95% CI, 0.915-1.874; P = 0.141).

The estimates for the overall survival (C) and leukemia-free survival (D) of patients in the development cohort who underwent peripheral blood stem cell transplantation (n = 427) were adjusted for the age at transplantation, sex, performance status, percentage of peripheral blasts, cytogenetic risk classification, response to chemotherapy, and times of transplantation. The survival curves were stratified according to the conditioning regimens. The hazard ratio for the overall survival of patients in the FLU+(BU or MEL) group versus those in the FLU+(BU or MEL)+(BU or MEL or CA or CY) group was 0.757 (95% CI, 0.528-1.086; P = 0.130); in the CA+CY+TBI group versus those in the FLU+(BU or MEL)+(BU or MEL or CA or CY) group was 1.022 (95% CI, 0.572-1.825; P = 0.942); in the BU+CY group versus those in the FLU+(BU or MEL)+(BU or MEL or CA or CY) group was 0.876 (95% CI, 0.549-1.396; P = 0.577). The hazard ratio for leukemia-free survival of patients in the FLU+(BU or MEL) group versus those in the FLU+(BU or MEL)+(BU or MEL or CA or CY) group was 1.032 (95% CI, 0.725-1.467; P = 0.863); in the CA+CY+TBI group versus those in the FLU+(BU or MEL)+(BU or MEL or CA or CY) group was 1.050 (95% CI, 0.610-1.807; P = 0.860); in the BU+CY group versus those in the FLU+(BU or MEL)+(BU or MEL or CA or CY) group was 1.126 (95% CI, 0.728-1.741; P = 0.595).

BMT, bone marrow transplantation; PBSCT, peripheral blood stem cell transplantation; CY, cyclophosphamide; TBI, total-body irradiation; BU, busulfan; MEL, melphalan; CA, cytarabine; FLU, fludarabine.
