# Supplementary material for: Personalized prediction of overall survival in patients with AML in non‐complete remission undergoing allo‐HCT
Source: Cancer Med. 2021 Jun 16;10(13):4250–68. doi: 10.1002/cam4.3920 (PMC8267144; doi:10.1002/cam4.3920)
Supplement: Supplementary file 2 — Table S1 [file CAM4-10-4250-s002.docx]

| **TABLE S1** Univariable analysis of the overall survival of patients in the development cohorts (*n* = 2534) | | | | | |
| --- | --- | --- | --- | --- | --- |
|  |  | **Cytogenetic risk classification** | **HR** | **95% CI** | ***P*** |
| **Cytogenetics** |  |  |  |  |  |
|  | normal | Favorable | 1.000 |  |  |
|  | trisomy8 | Favorable | 0.845 | 0.661-1.081 | 0.1799 |
|  | inv16 | Favorable | 1.053 | 0.545-2.034 | 0.8773 |
|  | other non-defined | Favorable | 1.087 | 0.943-1.253 | 0.2499 |
|  | t(9;11) | Favorable | 1.153 | 0.635-2.094 | 0.6392 |
|  | t(8;21) | Intermediate | 1.261 | 1.006-1.581 | 0.0446 |
|  | inv3, t(3;3) | Intermediate | 1.269 | 0.925-1.742 | 0.1402 |
|  | -7/del(7q) | Intermediate | 1.292 | 1.031-1.620 | 0.0264 |
|  | 11q23 other than t(9;11) | Intermediate | 1.322 | 1.041-1.678 | 0.0221 |
|  | complex | Intermediate | 1.519 | 1.330-1.734 | < 0.0001 |
|  | unevaluable | Poor | 1.832 | 1.396-2.405 | < 0.0001 |
|  | -5/del(5q) | Poor | 1.928 | 1.608-2.313 | < 0.0001 |
|  | -17 | Poor | 1.950 | 1.386-2.743 | 0.0001 |
|  | t(6;9) | Poor | 2.033 | 1.255-3.294 | 0.0039 |
|  |  |  |  |  |  |
| Abbreviation: HR, hazard ratio. | | | | | |
|  |  |  |  |  |  |
|  | | | | | |
