# Supplementary material for: Personalized prediction of overall survival in patients with AML in non‐complete remission undergoing allo‐HCT
Source: Cancer Med. 2021 Jun 16;10(13):4250–68. doi: 10.1002/cam4.3920 (PMC8267144; doi:10.1002/cam4.3920)
Supplement: Supplementary file 3 — Table S2 [file CAM4-10-4250-s003.docx]

| **TABLE S2** The details of ≥3-drug regimen, including fludarabine, for the treatment of patients in the development cohort who underwent cord blood transplantation | | | |
| --- | --- | --- | --- |
| **FLU+(BU or MEL)+(BU or MEL or CA or CY)** |  | ***n*** | **%** |
|  | FLU+BU+MEL | 125 | 34.9 |
|  | CA+FLU+BU+MEL | 76 | 21.2 |
|  | CA+FLU+BU | 76 | 21.2 |
|  | CA+FLU+MEL | 55 | 15.4 |
|  | FLU+BU+CY | 22 | 6.1 |
|  | CA+FLU+BU+CY | 4 | 1.1 |
|  | Total | 358 | 100.0 |
| Abbreviations: FLU, fludarabine; BU, busulfan; MEL, melphalan; CA, cytarabine; CY, cyclophosphamide. | | | |
|  |  |  |  |
|  |  |  |  |
