# Supplementary material for: Personalized prediction of overall survival in patients with AML in non‐complete remission undergoing allo‐HCT
Source: Cancer Med. 2021 Jun 16;10(13):4250–68. doi: 10.1002/cam4.3920 (PMC8267144; doi:10.1002/cam4.3920)
Supplement: Supplementary file 4 — Table S3 [file CAM4-10-4250-s004.docx]

| **TABLE S3** Distribution of scores in the validation cohorts based on the previous scoring system | | | |
| --- | --- | --- | --- |
|  |  | **Bone marrow transplantation** | **Peripheral blood stem cell transplantation** |
|  |  | **(*n* = 167); *n* (%)** | **(*n* = 93); *n* (%)** |
| **Scores** |  |  |  |
|  | 0 | 15 (9.0) | 6 (6.5) |
|  | 1 | 41 (24.6) | 21 (22.6) |
|  | 2 | 63 (37.7) | 26 (28.0) |
|  | ≥3 | 48 (28.7) | 40 (43.0) |
|  |  |  |  |
|  |  |  |  |
|  | | | |
